# Analysis of an Australian death database of people with intellectual disability living out of the family home: Place of death and associated variables

**DOI:** 10.1177/17446295231175541

**Published:** 2023-05-16

**Authors:** Michele Y. Wiese, Roger J. Stancliffe, Seeta Durvasula, Daniel W. Piepers, Nathan J. Wilson

**Affiliations:** School of Psychology, 371449 Western Sydney University, Australia; Sydney School of Health Sciences, Centre for Disability Research and Policy, 522555 The University of Sydney, Australia; Sydney Medical School, Centre for Disability Studies, 522555 The University of Sydney, Australia; School of Psychology, 371449 Western Sydney University, Australia; School of Nursing and Midwifery, 6489 Western Sydney University, Australia

**Keywords:** intellectual disability, place of death, nursing, disability support worker, dysphagia

## Abstract

This study reports on a five-year data set about the deaths of 599 individuals in New South Wales Australia, who at the time of their death were living in out-of-home care. Analysis aimed to: i) gain a clearer understanding of place of death for people with intellectual disability; and ii) identify and analyse associated variables to investigate how well they predict place of death for this population. Hospital admissions, polypharmacy and living situation were the strongest standalone predictors of place of death. A hospital death was more likely if the target population were subject to polypharmacy, lived in a group home, had a moderate intellectual disability or had GORD. Death, and place of death, is an issue requiring individual consideration. This study has identified some of the variables that need attention when supporting people with intellectual disability to have a good death.

## Background

Identifying where people die and the relationships between health and associated variables at the time of death informs health care policy and the allocation of resources for quality end-of-life care (Heslop & Hoghton, 2019). For the general community, this information has assisted the gradual move toward deaths that aim to balance expressed preferences of the individual dying, caregiver capacity, availability of both health and social supports, and flexible resources that match changing care needs, for example, transition from home to hospital if health needs necessitate. When this balance is achieved, a so-called ‘good death’ might be the outcome for all. Such a death is typically indicated by death at home or in a home-like environment, open awareness about the dying, knowledge to make informed decisions, respect, acceptance, and quality social and health care (Cottrell & Duggleby, 2016; Vanderpool, 2015). For people with intellectual disability, amongst the most vulnerable and marginalised in our society, the extent to which this is achieved remains unclear. A sensible first step to inform this quest is to determine where people with intellectual disability die, and the associated variables related to where death occurs.

Research comparing place of death of people with intellectual disability to the general community is beginning to emerge. Segerlantz et al. (2020), comparing the deaths of Swedish cancer patients aged 55 years and older showed that most with intellectual disability died in their group home or own home (51% of the sample; *n*=775), while most from the general population died in hospital (55% of the sample; *n*=2968). McCarron et al.’s (2017) comparison of deaths in Ireland showed a similar trend. For deaths of people with intellectual disability, 54% occurred in the usual home with 35% in hospital, while for the general community 48% occurred in hospital and just 25% at the usual home. Hunt et al.’s (2020) research from the UK broadly echoes these findings, however Brameld et al. (2018) did report that 47% of their Australian sample with intellectual disability died in hospital, although this study included people with intellectual disability living in aged care settings. To-date then, when only examining place of death as the key variable, available research suggests that people with intellectual disability more typically die in their usual home, while people from the general community more typically die in hospital.

Comparing populations about place of death alone though offers an incomplete picture, as intellectual disability only study samples illustrate. Todd et al. (2020) in a UK study of 202 decedents in accommodation settings showed slightly more died in hospital (49.5%) than the usual home (46.5%). Likewise, in research from New Zealand, for 62 reported deaths of people living in disability accommodation settings, 56% died in hospital and 37% in their usual home (Todd et al., 2021). Overall, the reported research examining place of death as a single variable shows contrasting results, likely explained partly by population and sampling differences, as well as differing end-of-life care practices in different residential settings. For example, Brameld et al.’s (2018) West Australian population-based study also included people with intellectual disability who lived in residential aged care. Some 23% of Brameld et al.’s (2018) decedents with intellectual disability died in residential aged care. When removing the *n*=136 who died in residential aged care from Brameld et al.’s *n*=591 sample of decedents with intellectual disability, leaves 278 of the remaining 455 who died in hospital, or 61.1%, in contrast with the 47% for Brameld et al.’s overall study, as noted above.

There are also additional variables at play, some of which add complexity rather than clarity. A few are briefly addressed here to illustrate. The evidence is clear, for example, that people with intellectual disability experience poorer health compared to the general community, and multiple complex health morbidities that increase with age (Hussain et al., 2020; Liao et al., 2021). While greater reliance on health services is also typical, the available research suggests a more complicated picture. McCarron et al. (2017) reported that deaths of people with intellectual disability were characterised by slightly higher rates of GP visits in the year preceding death, but lower hospital admissions compared to the general population. Segerlantz et al. (2020) found those with intellectual disability were less likely than the general population to have received palliative care in the year leading to death. Meanwhile, in Australia Brameld et al. (2018), with measures offering more detailed information, showed increased presentation to hospital emergency departments compared to the general population, but fewer hospital admissions. For those admissions that did occur, length of stay was longer, intensive care and ventilator support was more likely, but hospital-based palliative care was less likely compared to the general population. The interplay between place of death, pre-existing health comorbidities, health care utilisation, hospitalisations, and palliative care is not well understood, and likely multifaceted.

The extent to which the degree of intellectual disability acts as a contributing variable in this complex interplay is likewise unclear, perhaps complicated by the absence of, or variation in measurement of variables across published studies. In the research summarised thus far, the degree of intellectual disability was either not specified (Brameld et al., 2018; Segerlantz et al., 2020), or measured in such a way that comparison between like populations is limited. To illustrate, Hunt et al. (2020) measured support needed to complete key activities without help (e.g., getting dressed) while McCarron et al’s. (2017) research identified level of intellectual disability (e.g., moderate or severe) based on caregiver report. Intellectual disability was specified for both studies, but its bearing on where the person died was not analysed. As a result, the available research sheds little light on either the extent to which degree of intellectual disability influences where death occurs, or its possible mediator role with the other variables.

Place of death may also be reflected by the extent to which a death is expected, or not. Todd et al. (2020), in a UK study examining the deaths of people with intellectual disability in accommodation settings, showed that expected deaths, commonly involving cancer or dementia, were more likely to occur in the individual’s usual home. If, however, the deaths were unexpected, these were more likely to occur in hospital.

A growing body of research shows that a substantial proportion of unexpected deaths of people with intellectual disability could be avoided with timely access to health care (Brameld et al., 2018; Glover et al., 2017; Ombudsman New South Wales, 2018). These potentially avoidable deaths occur at higher rates for people with intellectual disability. Trollor et al. (2017) showed that 38% of deaths of people with intellectual disability were potentially avoidable, compared to 17% in a comparable general population. Brameld et al.’s (2018) research mirrored these findings. Whether unexpected deaths occur because of known inequities in health care access, symptom identification problems arising from communication difficulties common to intellectual disability, diagnostic overshadowing, extent of intellectual disability or any these together in complex relationship, is largely unknown. Further if, and how, these relate to place of death also remains unclear.

Place of death and the interplay of associated variables, some which may reflect inequity and some characteristic of the intellectual disability experience, is complex (Bernal et al., 2022). Against this backdrop, a unique opportunity to examine these issues in detail presented itself with the authors’ opportunity to analyse a data set comprising five consecutive years of information about deaths of people with intellectual disability living in out-of-family-home care in the state of New South Wales (NSW), Australia. This comprehensive data set enabled analysis of the relationship between place of death and a number of key health and service factors. The current study reports the findings of this analysis. Two aims underpinned the analysis:

1. Identify the place of death of people with intellectual disability who, at the time of death, had as their principal place of residence an accommodation support service funded or operated under the governance of the NSW state government.
2. Identify and analyse associated variables in relation to place of death, including health variables, level of intellectual disability, type of usual home at time of death, palliative care plan status, and hospitalisations in the year preceding death.

## Methods

This study used a retrospective descriptive design involving secondary analysis of a deidentified data set. As a non-identifiable data set, the project was exempt from Human Research Ethics Committee review in accordance with Section 5.1.22 of the Australian *National Statement on Ethical Conduct in Human Research* (National Health & Medical Research and Council Australian Research Council and Australian Vice-Chancellors' Committee, 2007 and amendments 2009).

### Context and the data set

At the time of the data reported here the NSW state government was responsible for funding and regulating most disability services in the state, including accommodation services for individuals with disability who lived out of the family home. Accommodation types included group homes, large residential centres, specialist supported living, small residential, drop-in support, and other, all of which were operated by either the government itself or not-for-profit, non-government organisations, as well as assisted boarding houses run by a small for-profit sector. Group homes typically have between three and five residents with 24-hour staff support, large residentials are the few remaining institutional complexes in NSW, specialist supported living is for those with specific complex needs, small residentials are purpose-built facilities for approximately 10 people with very high support needs, drop-in support is as stated, other includes respite facilities, and boarding houses are room-to-rent services with limited staff support. The data set reported here, known as the Client Death Notification (CDN) database, was administered by the NSW state government through its Family and Community Services (FACS) department. The CDN database comprises five consecutive years of high quality, detailed data about the deaths of *all* people with intellectual disability who at the time of their death were residing in these accommodation types during the period 2012 to 2016 inclusive. Individuals with intellectual disability residing permanently in residential aged care facilities during that period were not included in the database as their accommodation was managed by a separate Federal government department.

The CDN database drew data from the Client Death Notification (CDN) Form (NSW Government Family & Community Services Ageing Disability & Home Care, 2016), a paper questionnaire requiring mandatory submission to FACS when a client dies. The CDN form includes information on the following items: personal details, details about the death, accommodation at the time of death, nature of the person’s disability, swallowing, breathing and choking risks, lifestyle risks, other health issues, medication and consent, summary of health providers, details about problem behaviours, illnesses, number of hospitalisations, serious injuries and falls that occurred in the 12-months prior to the death, and any police notifications of the death.

Received by the authors as an excel spreadsheet in April 2017, the CDN database summarised 607 individual deaths between 2011 and 2016. This spreadsheet did not include personal, identifying details. The 2011 data were incomplete so the number of reported deaths from 2011 was small (*n*= 8). Therefore, these were excluded from analysis, leaving a dataset of 599 reported deaths for the 5 years from 2012 to 2016 inclusive. For each reported death the database comprised 175 possible items. Across the 599 deaths, an average of 74 complete data items were recorded per individual (range 48-120; *SD* = 17.63). Data cleaning involved recoding variables where the language in variations of the CDN form changed over time and where string data required categorisation for statistical analyses.

### Data analysis

The dataset was transferred to SPSS for descriptive and inferential analyses. Descriptive analysis involved categorical data analysed as frequencies and proportions, with continuous data reported as means and standard deviations. Inferential analyses included chi-square and logistic regression with place of death (hospital or not) as the dependent variable. Bonferroni correction was applied to the bivariate analyses presented in Table 2 as there were 22 separate chi-square analyses; statistical significance was therefore set at *p*<0.0023.

## Results

Across 5 years of data, the mean number of deaths was 120 per year (range 107-136). Most people died in hospital (*n*=354, 59.1%), followed by death in other places such as the person’s home (*n*=209, 34.9%), at friend’s/family home (*n*=14, 2.3%), at an aged care facility (*n*=4, 0.7%), or when transiting to a hospital or GP (*n*=4, 0.7%). The number of expected deaths (data recorded only for years 2013–2016; *N*=488), was slightly lower (*n*=236, 48.4%) than the number of unexpected deaths (*n*=252, 51.6%). The demographic and health profiles of all people who died are presented in Table 1. The mean age at time of death was 54.2 years (range 10-90) with the largest proportion of deaths occurring in the 55-64 years category. Dysphagia (swallowing difficulties) and needing mealtime assistance were the commonest reported health conditions/health risks, followed by epilepsy, recurrent respiratory infections, and gastro-oesophageal reflux disease (GORD). Polypharmacy, defined as 5 or more concurrent medications (Masnoon et al., 2017), was prevalent across the data set (76% of the total sample).

**Table 1.** Demographic and health profile (*N* = 599).

Variable	Frequency (*n*)	Percent (%)
Level		
Age group (years)		
0–24	18	3.0
25–34	42	7.0
35–44	72	12.0
45–54	154	25.7
55–64	166	27.7
65–74	106	17.7
75 and older	41	6.8
Sex		
Female	248	41.4
Male	351	58.6
Level of Intellectual Disability		
Mild	83	13.9
Moderate	145	24.2
Severe	166	27.7
Profound	46	7.7
Unknown/not reported	159	26.6
Health condition/health risk		
Asthma	92	15.4
Cancer	119	19.9
Diabetes	93	15.5
Epilepsy	262	43.7
GORD	220	36.7
High BP	139	23.2
Mealtime assistance	287	47.9
Osteoporosis	174	29.0
Recurrent respiratory infections	222	37.1
Dysphagia	348	58.1
Tube feeding	71	11.9
Number of prescribed medications^a^		
0–2	59	12.1
3–8	217	44.5
9–13	123	25.2
14–19	68	13.9
20–28	17	3.5
28+	4	0.8
Living situation		
Group home	284	47.4
Large residential	158	26.4
Specialist supported living	54	9.0
Small residential	38	6.3
Drop-in support	29	4.8
Assisted boarding house	23	3.8
Other	13	2.2
Governance		
Government operated service	284	47.4
Non-government operated service	292	48.8
Assisted boarding house	23	3.8

^a^Data only available for the years 2013–2016 (*N* = 488).

Data for hospital admissions, serious injuries and falls in the previous 12 months were reported for deaths in years 2015-2016 (*N*=238). A large proportion reported at least one hospital admission (*n*=187, 78.5%) in the 12 months prior to death, with 26.5% (*n*=63) having two, and 19.2% (*n*=46) having three or more hospital admissions. A total of 35 (14.7%) had a serious injury reported in the previous 12 months, the majority of which were fractures (*n*=20). Eighty two (34.5%) had at least one fall in the previous 12 months.

### Place of death bivariate analyses

Bivariate analyses explored relationships between place of death as a dependent variable, using other key variables as the independent variable; see Table 2. Events 12-months prior to death showed that few (16.7%) with no hospital admissions in the previous 12-months died in hospital, whereas 68.7% with 1 or more admissions in the last year had hospital deaths. People with polypharmacy (defined as being prescribed five or more routine medications) were also more likely to die in hospital (62.9%). Of note was that people living in group homes were more likely to die in hospital (69.7%) than those who lived in large residential centres (48.1%).

**Table 2.** Bivariate analyses of relationship between place of death in hospital (Y/N) and selected variables (*N* = 599).

Variable type	% died in hospital	χ^2^	p
Variable category			
Level			
Demographics			
Age group		6.55	0.365
Sex		0.34	0.562
Level of intellectual disability		6.40	0.094
Health condition/risks			
Asthma		0.007	0.932
Cancer		3.26	0.071
Diabetes		0.49	0.486
Epilepsy		0.23	0.634
GORD		5.01	0.025
High BP		0.58	0.448
Mealtime assistance		0.78	0.377
Osteoporosis		0.001	0.975
Recurrent respiratory infections		1.80	0.179
Dysphagia		0.06	0.804
Tube feeding		1.67	0.197
Events 12 months prior to death^a^ (*N* = 238)			
Falls		0.39	0.697
Hospital admissions		50.002	<0.001
0	16.7_a_		
1	66.7_b_		
2	73.0_b_		
3 or more	78.3_b_		
Illnesses		0.02	0.898
Injuries		0.54	0.462
Polypharmacy – 5 or more medications^b^(*N* = 488)		16.79	<0.001
No	41.4_a_		
Yes	62.9_b_		
Expected death^b^(*N* = 488)		3.12	0.078
Service system			
Governance		5.38	0.068
Living situation		34.30	0.001
Group home	69.7_a_		
Large residential	48.1_b_		
Specialist supported living	55.6_ab_		
Drop-in support	27.6_b_		
Assisted boarding house	60.9_ab_		
Small residential	57.9_ab_		
Other	46.2_ab_		

*Note*. Bonferroni corrected significance was set at *p*<.0023. Percentage died in hospital only reported for variables that were significantly associated with this outcome.

^a^2015–2016 data.

^b^2013–2016 data; all other rows 2012–2016 data.

a,b Within each variable category, each subscript letter denotes a subset of levels that did not differ significantly at *p*<.05, Bonferroni corrected. These post-hoc comparisons were only conducted for analyses where the overall result met the Bonferroni corrected criterion of *p*<.0023.

### Presence of palliative care plan bivariate analysis

Having a palliative care plan was not associated with place of death (see Table 3). People were significantly more likely to have a palliative care plan if their death was expected (70.3%) or if they had a cancer diagnosis (62.2%).

**Table 3.** Comparisons of presence of a palliative care plan by selected variables.

Variable	Palliative care plan				χ^2^
	No		Yes		
	*n*	%	*n*	%	
Died in hospital (*N* = 599)					1.44
No	166	67.8	79	32.2	
Yes	223	63.0	131	37.0	
Expected death (*N* = 489)^a^					176.54***
No	224	88.5	29	11.5	
Yes	70	29.7	166	70.3	
Cancer (*N* = 599)					48.00***
No	344	71.7	136	28.3	
Yes	45	37.8	74	62.2	

*Note*. Row percentages shown.

^a^2013–2016 data.

***p<.001.

### Multivariate analysis

Logistic regression of place of death (hospital or not) was undertaken and is reported in Table 4. With one exception (hospital admissions), we selected independent variables that achieved *p*<.10 as reported in Table 2. Although significant under univariate analysis (Table 2), hospital admissions in the last year was not used in the logistic regression analysis because these data were only available for 2015-16 and would have more than halved the overall sample size, with consequent loss of statistical power and representativeness.

**Table 4.** Logistic regression analysis of place of death (hospital or not) (*N* = 359).

Predictor (reference category)	Wald	Sig.	OR	95% CI for ORs	
				Lower	Upper
Living situation (large residential)	20.046	.003			
Group home	14.415	.000	3.246	1.768	5.962
Specialist supported living	3.770	.052	2.321	.992	5.428
Drop-in support	.000	.995	1.004	.263	3.831
Assisted boarding house	3.341	.068	3.933	.906	17.078
Small residential	.444	.505	1.489	.462	4.796
Other	.157	.692	1.409	.258	7.688
Governance (govt. operated)	1.947	.163	1.558	.836	2.903
Level of intellectual disability (mild)	4.818	.186			
Moderate	4.578	.032	2.102	1.064	4.151
Severe	2.959	.085	1.929	.913	4.076
Profound	1.054	.305	1.693	.620	4.625
GORD (no)	4.904	.027	1.752	1.067	2.877
Cancer (no)	2.201	.138	1.598	.860	2.967
Polypharmacy^a^ (no)	3.936	.047	1.761	1.007	3.080
Expected death^a^ (no)	1.437	.231	1.350	.827	2.203

*Note*. Dependent variable coding: hospital death = 1, non-hospital death = 0.

^a^based only on 2013–2016 data.

Nagelkereke *R*^2^ = .15.

As Table 4 shows, living situation remained significant overall (*p* = 0.003), with comparisons showing that people with intellectual disability living in group homes were over three times as likely to die in hospital compared to those who lived in a large residential centre. Level of intellectual disability and having GORD were also significant, where having moderate intellectual disability or having GORD each meant being more likely to die in hospital. People with polypharmacy were 1.76 times as likely to die in hospital.

## Discussion

The findings of this study add to the existing research about place of death and related variables for people with intellectual disability living out of the family home at the time of their death. A select few key findings are taken up for discussion and situated within the available literature.

## Place of Death

First to the key outcome measure, place of death. The finding that hospital was the most common place of death for people with intellectual disability offers an alternative picture to the previously summarised research comparing intellectual disability to the general population on place of death (Brameld et al., 2018; Hunt et al., 2020; McCarron et al., 2017; Segerlantz et al., 2020). The previous research suggested that on the place of death variable alone in comparison to a like general population, it is more common for people with intellectual disability to die in their usual home. The findings reported here, however, align with the general population findings, for whom hospital deaths are more typical. The place of death data in this study does though align with other research exploring the detail of deaths of people with intellectual disability without a general population comparison group (e.g., Todd et al., 2020; 2021) or when excluding deaths in aged care settings (Brameld et al., 2018) where the results (61.1%) were very similar to the 59.1% we reported. In addition to sampling issues, it is also likely that, as indicated in the Introduction, there are additional variables in play that were not able to be explored with the available variables in the dataset. For instance, the mandatory nature of reporting resident deaths to the coroner in NSW may mean that some disability services view such reporting as a service or client-related risk, and therefore send dying clients elsewhere (such as to hospital or residential aged care). This issue may have affected our findings on place of death in unknown ways, but in the absence of available data these possible influences remain open to conjecture.

The proposal that a range of additional variables likely relate to where death occurs has been supported in our findings. The bivariate analyses (Table 2) showed three key variables that bear a significant relationship: hospital admissions, polypharmacy, and living situation at the time of death. With respect to hospital admissions, individuals with one or more hospital admissions during the year preceding death were more likely to die in hospital, and conversely very few with no hospital admissions subsequently died in hospital. This might at first glance seem logical - repeat hospitalisations in the year prior to death suggest the presence of serious illness, and the available research does show higher incidence of serious health conditions as characteristic of intellectual disability (Liao et al., 2021). Serious illness and repeat hospitalisations might logically translate to greater chance of hospital death. However, the findings pertaining to expected deaths, discussed below, suggest an additional explanation. Over half of the reported deaths were unexpected, suggesting that for a substantial proportion of deaths, prior serious illness that might ultimately result in death was not in fact identified by caregivers. This explanation has important implications for caregiver qualifications and skill set.

Polypharmacy was also related to place of death, with those receiving five or more medications being 1.76 times as likely to die in hospital. This finding is not surprising when added to the evidence previously discussed about serious health conditions and hospitalisations – all together suggesting complex healthcare needs in the last year of life. Previous research showed the positive correlation between number of daily medications and chronicity associated with complex health and disability (Wilson et al., 2020). While concurrent medication use might be an expected product of the health comorbidities common to intellectual disability, this often escalates with even greater medication use as a person deteriorates, with the added health consequences, including side effects and drug interactions, adding further complexity to the person’s care needs. This highlights the imperative for careful day-to-day assessment and oversight by skilled staff, such as registered nurses, with collaborative monitoring and review by the medical prescribers and the pharmacists who dispense the medications, in the year preceding death.

Of interest in the current study is the finding that living situation at the time of death was related to where death occurred. Those whose usual living situation was a group home were over three times more likely to die in hospital compared to those living in large residential centres at the time of their death. This variation could be explained at least in part by the profile of staff qualifications and skills at the respective usual living situations. At the time of the CDN database, large residential centres in NSW employed registered nurses as a part of the care cohort. Group homes meanwhile, employed disability support workers whose qualification did not mandate and rarely included nurse training. Life-limiting illness and approaching death in large residential centres may have been ably managed by day-to-day oversight by registered nurses whose professional training and advanced decision-making skills meant competence to provide intensive health related supports (Wilson et al., 2020). Faced with similar conditions in group homes, disability support workers may not have had the necessary skills to manage deteriorating health as death approached, so the likelihood of transfer to hospital may have increased.

To the best of the authors’ knowledge, this study is the first to provide evidence of a relationship between place of death and level of intellectual disability. Individuals with a moderate intellectual disability were over two times as likely to have a hospital death compared to those with mild intellectual disability. The interpretation of finding is unclear but does suggest the relationship between degree of intellectual disability and other variables warrants future detailed investigation.

GORD was significantly associated with hospital death. Along with previous research (Durvasula & Beange, 2001; van Timmeren et al., 2017), this study has identified GORD as a prevalent health condition common to the experience of intellectual disability, so perhaps its role in place of death is not surprising. Of concern though is the existing evidence indicating that untreated reflux when co-occurring with dysphagia can result in recurrent respiratory tract infections, a known leading cause of death for this population, and one which is usually amenable to treatment and thereby potentially avoiding death (Brameld et al., 2018; Landes et al., 2021; Trollor et al., 2017). That being said, regardless of medical treatment, skilled staff are still required to identify and manage the myriad of daily issues associated with dysphagia, in particular when aspiration of food and/or fluid is silent. A recent literature review noted poor identification and management of emerging and acute health needs, such as dysphagia, choking/aspiration and respiratory supports within disability services (Salomon & Trollor, 2019).

The findings on the existence of a palliative care plan at time of death are noteworthy. Although the existence of a palliative care plan bore no relationship to where a person died, individuals were significantly more likely to have a palliative care plan if their death was expected **or** if the health condition/ health risk at the time of death was cancer. Taken together, these findings suggest some relationship between expectation of death as well as diagnosis at the time of death. Palliative care research in intellectual disability has often been cancer focused (Gomes et al., 2010; Segerlantz et al., 2020; Tuffrey-Wijne et al., 2008; Tuffrey-Wijne et al., 2007), with perhaps less attention given to other known diagnoses. A noted gap in the palliative care research are supports for younger people with multiple chronic and complex health needs who are now living into their teens and twenties, for whom death would have been in infancy or childhood in previous decades. This dominant focus on palliation for people with cancer is also likely a by-product of contemporary palliative care with its historical routes in cancer care, which has only in recent years taken a broader approach inclusive of all life-limiting illnesses (Allen et al., 2018; World Health Organization). In reference to our findings, it is possible that caregivers and health professionals alike have responded to the more well-known and obvious cancer health risk, with palliative care plans instituted accordingly. The outcome of this focus, however, is the likelihood of disadvantaged deaths for those with life-limiting illnesses that are not cancer-related, but equally amenable to treatment and potentially death avoidable (Hosking et al., 2016).

Several additional findings are worthy of highlighting. The number of expected deaths (48.4%) was slightly lower than unexpected deaths (51.6%). These findings echo other research, indicating that a substantial proportion of deaths of people with intellectual disability come as a surprise (Bernal et al., 2021; Todd et al., 2020). Of particular interest in the present study is that although this collection of findings may illustrate inequities in access to health care (Hunt et al., 2020), expected death does not appear to have a significant relationship with place of death.

The age at death finding (54.2 years) from the CDN database is marginally younger than reported in other research involving adults with ID who use ID services. For comparison, Todd et al. (2020) found a mean age at death of 56.8 years in the UK, Todd et al. (2021) indicated mean age at death of 61.2 years in New Zealand, and Trollor et al. (2017) showed median age of 54 years in Australia. Although these data reflect some variation, taken together they align with the conclusion that deaths of people with intellectual disability indicate a “young to middle aged group” (Todd et al., 2021, p. 300). Variations could be attributable to a range of factors including level of intellectual disability and health risks, both known to bear relationship to age of death (Liao et al., 2021; O’Leary et al., 2018). More importantly, our reported add evidence to the international research showing that people with intellectual disability die at a younger age than do those in the general population (Dieckmann et al., 2015; Glover et al., 2017; Lauer et al., 2015; Trollor et al., 2017).

With our focus on place of death as a key outcome variable, this should not be interpreted as suggesting where death *should* occur. As proposed in the Introduction, a so-called ‘good death’ might result when the combination of death awareness, honouring preferences, quality social and health care, and flexibility, all work in unison for the person with intellectual disability and their caregivers facing oncoming death. It is important to acknowledge that the CDN database included no information on the person’s expressed preferences about place of death. Two small-scale studies have shown that when asked, and like a comparable general population, people with intellectual disability express a preference to die at home (Hunt et al., 2020; McCarron et al., 2017). Other research offers a reminder, however, that while honouring the expressed preference about place of death is an important indicator, it should not be the only one, particularly if it is prioritised at the expense of other quality indicators so important in end-of-life care (Segerlantz et al., 2020; Tuffrey-Wijne, 2009).

Some study limitations are noteworthy. The CDN database findings only involve to those who at their death lived in out-of-family home care. The findings are therefore not generalisable to deaths of residents from other settings, like family homes or residential aged care. However, the data set did offer detailed data across multiple variables about a large number of deaths, enabling a nuanced understanding about the place of death and associated variables.

For the future, preferred place of death is an obvious research gap, as is the extent to which the stated preference can be accommodated by caregivers. Regionality is another key variable for a future study as it would enable a rural / urban comparison and may shed some light on whether distance and service availability disparities influence where people with intellectual disability die. Staff skillset remains an under-reported variable that must be included in future studies to better understand how preferences can be supported, or potentially denied, if the caring for a person who is dying is beyond the scope of practice for disability support workers. Likewise, investigating the availability of highly skilled in-home care from registered nurses could relate to the capacity to honour place-of-death preferences. In the Australian context, how this might be funded remains unclear (Wilson et al., 2021). Although the Australian disability funding landscape has altered significantly since these data were collected, from state facility-based funding to federal individualised funding under the National Disability Insurance Scheme (NDIS), service providers still have responsibility for client death reporting, so these results remain highly relevant.

## Conclusion

Variation in place of death is a reminder that where people with intellectual disability die is a result multiple contributing variables, some distal, like pre-existing level of intellectual disability, and some more immediate, including health-care variables like hospitalisations and medication use, as well as living situation at the time of death. The data reported in this study, together with the previous research suggests that place of death is not consistently predictable, and perhaps nor should it be. Death, including where it happens, is an individual experience requiring individual care. This study’s contribution is to identify at least some of the individual variables that require attention such that something approximating the so-called ‘good death’ might be the outcome.
